# Home Telemonitoring Technology for Patients With Heart Failure: Cost-Consequence Analysis of a Pilot Study

**DOI:** 10.2196/32147

**Published:** 2022-06-02

**Authors:** Glory Apantaku, Craig Mitton, Hubert Wong, Kendall Ho

**Affiliations:** 1 School of Population and Public Health University of British Columbia Vancouver, BC Canada; 2 Digital Emergency Medicine University of British Columbia Vancouver, BC Canada

**Keywords:** cost-consequence analysis, feasibility study, pilot study, heart failure, cardiology, cardiovascular disease, economic analysis, telehealth, health care cost, home monitoring, digital monitor, health monitor

## Abstract

**Background:**

Heart failure (HF) is a costly health condition and a major public health problem. It is estimated that 2%-3% of the population in developed countries has HF, and the prevalence increases to 8% among patients aged ≥75 years. Home telemonitoring is a form of noninvasive, remote patient monitoring that aims to improve the care and management of patients with chronic HF. Telehealth for Emergency-Community Continuity of Care Connectivity via Home-Telemonitoring (TEC4Home) is a project that implements and evaluates a comprehensive home monitoring protocol designed to support patients with HF as they transition from the emergency department to home.

**Objective:**

The aim of this study is to assess the cost of using the home monitoring platform (TEC4Home) relative to usual care for patients with HF.

**Methods:**

This study is a cost-consequence analysis of the TEC4Home pilot study. The analysis was conducted from a partial societal perspective, including direct and indirect health care costs. The aim is to assess the costs of the home monitoring platform relative to usual care and track costs related to health care utilization during the 90-day postdischarge period.

**Results:**

Economic analysis of the TEC4Home pilot study showed a positive trend in cost savings for patients using TEC4Home. From both the health system perspective (Pre TEC4Home cost per patient: CAD $2924 vs post TEC4Home cost per patient: CAD $1293; *P*=.01) and partial societal perspective (Pre TEC4Home cost per patient: CAD $2411 vs post TEC4Home cost per patient: CAD $1108; *P*=.01), we observed a statistically significant cost saving per patient.

**Conclusions:**

In line with the advantages of conducting an economic analysis alongside a feasibility study, the economic analysis of the TEC4Home pilot study facilitated the piloting of patient questionnaires and informed the methodology for a full clinical trial.

## Introduction

Heart failure (HF) is a costly health condition and a major public health problem. An estimated 2%-3% of the population in developed countries has HF. The prevalence increases to 8% among patients aged ≥75 years [1]. Although it is the common final stage of many heart diseases, its manifestations can be difficult to diagnose accurately [1]. According to clinical criteria established by the Framingham Heart Study, a diagnosis of HF is confirmed when two major criteria such as elevated jugular venous pressure, pulmonary rales, or a third heart sound are found, or when one major criterion and two minor criteria, including peripheral edema, dyspnea on exertion, or hepatomegaly, are confirmed [1]. A diagnosis of HF carries substantial risk of morbidity and mortality, despite advances in management.

Home telemonitoring is a form of noninvasive, remote patient monitoring that has gained attention as a promising strategy for improving the care and management of patients with chronic HF. It can be particularly helpful for older adults and those who are frail as well as those at high risk of deterioration [2]. It involves the use of electronic devices and telecommunication technologies (eg, monitoring devices, handheld or wearable technologies, and intelligent sensors) for the digital transmission of physiological and other disease-related data from the patient’s home to a health care center providing care and clinical feedback, enabling the collection of clinical data remotely on a regular basis. Using home monitoring technology can result in early detection of clinical decompensation in patients with HF, making it possible to provide timely intervention to prevent mortality events or further deterioration of the patient’s condition [2]. Research has shown that for people with HF, structured telephone support and noninvasive home telemonitoring reduces the risk of all-cause mortality and HF-related hospitalizations [3]. These interventions have also been demonstrated to be a major factor in improved self-care behaviors and health-related quality of life and HF knowledge improvements [3].

Telehealth for Emergency-Community Continuity of Care Connectivity via Home-Telemonitoring (TEC4Home) is a project that will implement and evaluate a home monitoring protocol designed to support patients with HF as they transition from the emergency department (ED) to home. The system uses home health monitoring technologies procured by TELUS Health to collect biometric measurements (ie, weight, blood pressure, pulse, oxygen saturation), which feed the monitoring software data to monitor and surveil patient deterioration in an effort to avoid unnecessary ED visits and hospitalizations. In addition, patients are provided a tablet to answer questions on how they feel [4]. TEC4Home has three broad aims. The first aim is to decrease 90-day readmission rates and improve clinical outcomes by increasing the safety and quality of care for patients with HF at home after discharge from the ED [4]. Second, the program aims to help increase patients’ engagement and understanding of their condition; the system also aims to improve communication and continuity of care during the transition from ED to home [4]. Finally, TEC4Home aims to achieve a reduction of resource utilization (eg, ED visits and readmissions) to achieve cost savings for the health care system [4].

The aim of this study is to assess the cost of implication of using the home monitoring platform (TEC4Home) relative to usual care and further track costs related to all utilization during the 90-day postdischarge period. Studies have shown that decision makers desire a disaggregated presentation of study costs and outcomes (consequences), which could include changes in survival, quality of life, or indicators of patient satisfaction [5,6]. As such, in this study, a cost-consequences approach was taken. Cost-consequence analysis (CCA) has been defined as an analysis in which costs and effects are calculated but not aggregated into quality-adjusted life years or cost-effectiveness ratios [7]. A CCA that involves comparing the costs and outcomes associated with the home monitoring intervention is more appropriate than a full economic evaluation because this is a pilot study with a limited sample. This form of analysis allows decision makers to compare explicitly the costs associated with usual care and home monitoring technology with the outcomes studied in this pilot. A health system and partial societal perspective was chosen to evaluate the cost-consequence of TEC4Home (ie, direct costs within the health care system and out-of-pocket costs incurred by the patients). This approach was driven by the fact that patient costs and costs outside health care are relevant when it comes to the wider societal impact of this type of technology. This study evaluates the cost implications of the TEC4Home telemonitoring technology on the health system and patients.

## Methods

### Identification of Outcomes of Interest

The outcomes of interest included in the evaluation are quality of life, mortality, event rates, and costs. Event rates include visits to the ED, general practitioner (GP), or hospital, as well as hospital admissions and length of hospital stay. These outcomes are distinguished by events related to HF and events related to any cause (Textbox 1).

**Disaggregation of the outcome (event rates and costs related to health care utilization).**

**Event rates**

- Number of general practitioner visits
- Number of specialist outpatient visits
- Number of emergency department visits (all-cause)
- Number of hospital admissions and length of hospital stay (all-cause)

**Cost components related to health care utilization**

- General practitioner visits
- Specialist visits
- Emergency department visits
- Length of hospital stay
- Professional household care, personal care, physiotherapy, and mental health care–related visits (captured as part of out-of-pocket cost)

Cost components related to health care for patients with HF were determined by the TEC4Home trial. Direct costs within health care are derived from those cost components. A distinction was made between costs related to the intervention (including equipment costs, lease costs, and connection fee) and costs related to health care utilization (Textbox 1).

### Data Analysis

In this study, utilization includes hospital admissions, ED visits, community family physician visits, and other health provider visits. Utilization was captured through a simple resource utilization questionnaire administered to patients at the time of patient outcome data collection. For the CCA, outcomes are reported in natural units, such as the number of ED visits avoided. Costs are reported in monetary units and consideration is given to costs incurred by the health system and/or the individual patients enrolled in the study. The costs included are those associated with health system resource use, such as ED visits, specialist visits, and nights in hospital.

With each patient serving as his/her own control, we compared health care utilization 90 days before index admission to 90 days posttelemonitoring. The costs of the home monitoring platform relative to usual care were assessed and the study further tracked costs related to all utilization during the 90-day postdischarge period. A 2-tailed paired sample *t* test was used to compare the difference in cost observed between the pre and post periods.

The CCA compared the costs (such as treatment and hospital care) and the consequences (such as health outcomes) of TEC4Home with the standard care patients received before enrolling in the study (which involves clinic visits for clinical examination, assessment of signs and symptoms, assessment of medication use, and provision of self-care instructions).

## Results

From October 2016 to June 2017, a total of 519 patients were screened, and 70 patients were enrolled. Patients were excluded if they were unable to complete study procedures, were unable to access a nurse or technology, were having a coronary or structural heart intervention during admission, or had an anticipated survival of less than 90 days. Participants’ median age was 75 (range 43-97) years. Complete self-reported health care utilization data from before and after TEC4Home were available for 30 patients; the CCA is based on this data.

The CCA showed a significant reduction in cost associated with length of stay during hospital admission after TEC4Home. With regard to cost associated with ED visits, GP visits, and specialist visits, there was a cost reduction for patients in the home telemonitoring arm; however, this difference was not statistically significant (Table 1).

Additionally, there was a reduction in out-of-pocket costs for patients in the telemonitoring arm; however, this difference was not statistically significant. Patient self-reports on special costs related to their health condition (including drugs, aids to daily living, housekeeping or home care, or transportation to/from medical appointments) showed that patients enrolled in the TEC4Home program saved an average of CAD $118. Note that all dollar values presented in the manuscript are given in Canadian dollars. A currency exchange rate of CAD $1=US $0.78 is applicable.

**Table 1:** Aggregate health care utilization cost.

	Pre TEC4Home cost (mean), CAD $	Post TEC4Home cost (mean), CAD $	Cost reduction (95% CI) per patient, CAD $	*P* value
Emergency department visit cost^a^	618	262	–87 to 799	.11
General practitioner visit cost^b^	126	129	–52 to 47	.92
Length of stay cost^c^	10,792	3091	3772 to 11,631	<.001
Specialist visit cost^d^	160	132	–72 to 128	.57
Out-of-pocket cost	357	185	–49 to 395	.12

^a^Standard outpatient cost per the Canadian Institute for Health Information: CAD $314.15.

^b^General practitioner visit cost from the Ministry of Health Medical Services Commission payment schedule.

^c^Per diem ward (one night in hospital) per the Canadian Institute for Health Information: CAD $1520.20.

^d^Specialist visit cost from the Ministry of Health Medical Services Commission payment schedule.

From the health system perspective, which was calculated using patient self-report surveys on nights spent in hospital, ED visits, specialist visits, GP visits, other health professional visits, and average cost for TEC4Home (this includes home health monitoring deployment and cost of monitoring nurse), we observed a statistically significant cost saving per patient. In addition, analysis from the partial societal perspective, which included direct and indirect health care costs, showed a statistically significant cost saving per patient. Table 2 shows a breakdown of mean cost from the health system and societal perspective; the relatively wide 95% CIs speak to the small sample size and limited precision in this study.

**Table 2:** Health care utilization cost from health system and partial societal perspective.

Perspective	Pre TEC4Home cost per patient (CAD $)	Post TEC4Home cost per patient (CAD $)	Cost reduction (95% CI) per patient (CAD $)	*P* value
Health system	2924	1293	1631 (292-2324)	.01
Partial societal	2411	1108	1303 (266-1896)	.01

## Discussion

### Principal Findings

This CCA showed positive trends in cost savings in the TEC4Home pilot study. Analysis from the health system perspective and the partial societal perspective showed statistically significant cost savings for patients enrolled in the TEC4Home arm. Compared to the 3-month period prior to a patient’s index admission, health care utilization in the 3-month period postdischarge was statistically significantly lower for mean length of hospital stay. As a result of their reduced health care utilization, which was mainly driven by reductions in length of hospital stay, patients in the telemonitoring arm cost the health system less than their counterparts in the usual care arm. These patients also had lower out-of-pocket costs than those in the usual care arm.

The development of home telemonitoring technologies can be linked to an improved understanding of the role that early recognition of warning signs of clinical deterioration and responding appropriately in hospital intensive care units play in preventing serious adverse events [8-11]. Home telemonitoring technology targets patients with chronic conditions who have more frequent interactions with the health care system and are thus more exposed to the risk for adverse events. The relatively older skew of the sample in this pilot study reflects that reality. It brings care directly to patients’ homes to prevent hospitalization, improve their feelings of safety, and empower them to manage their chronic conditions [11]. The cost of adverse events is a burden on the health care system. The estimated economic burden of preventable adverse events in Canada in 2009-2010 was CAD $397 million. This estimate does not include additional costs incurred by the patients after discharge or costs associated with loss of productivity [12].

Although some studies have measured cost effectiveness, cost utility, and cost benefits of telemonitoring technologies in patients with HF, those studies were done alongside clinical trials. Conducting an economic analysis alongside a feasibility study is often not done, but it is useful in determining the main cost-driving events related to the technology being assessed [13]. It also facilitates the piloting of patient questionnaires to test for clarity and ease of use, ensure pivotal economic data is collected effectively, and estimate completion rates [13]. Furthermore, it provides insight into the sustainability of providing a service like telemonitoring and how such a service can be funded. This study was conducted in recognition of the importance of conducting analysis of relevant data at each point in the development and testing of interventions like home telemonitoring [13].

In line with the advantages of conducting an economic analysis alongside a feasibility study, this study facilitated the piloting of patient questionnaires and informed the methodology for the full clinical trial. The clinical trial has highlighted cost drivers that were not addressed by this pilot study. For example, the clinical trial now includes prescription drug costs using data from PharmaNet, which is a provincewide network that links all British Columbia pharmacies to a central data system and provides information on every prescription dispensed in community pharmacies. Additionally, the clinical trial includes administrative data collected from all sites to ensure improved accuracy in the measurement of all health utilization variables.

Successful use of telemonitoring technology is not based solely on the efficacy of the technology—rather, it is the result of integration of the technology and existing work practices of patients and clinicians who interact daily with the technology. The presence or absence of successful integration may result in differential technological performance [14-16]. Patient self-care has been identified as a key component of daily HF management [17]. In the adoption of these monitoring technologies, it is important for policy makers to carefully consider how the integration of telemonitoring with existing care management processes may create a need for modifications to existing practices and relations between various health professionals [14]. Policy makers also need to be aware of possible change management costs that come with adopting these technologies. To ensure telemonitoring is cost-effective and clinically effective, it is advisable that there is an effective alignment of proposed technologies with existing practices to facilitate a seamless connection among the various practices, especially in cases where there is a complex organizational setting [14].

Given the rising cost of health care, health planners are looking for alternative methods to provide care to patients that reduce pressure on the health budget while ensuring patients still get high-quality care; one such potential method is telemonitoring. In designing clinical trials to study the effect of these technologies, it is important to ensure the relevant study period is driven by clinical data as this would provide an improved understanding of the role these technologies can play in patient care. These relevant study periods should also drive the cost analyses that are conducted alongside these trials to evaluate the economic implications of adopting these technologies. Additionally, more studies need to adequately evaluate some less obvious costs related to remote monitoring such as database maintenance costs, technical support costs, and possible increases in health care resource use in response to alerts by the monitoring system.

### Limitations

There are a number of limitations in this study. The sample size for the TEC4Home feasibility study was relatively small and there may be systematic differences between the patients who were able to sign up for the study and adhere to the monitoring protocol and those who were not (eg, the former may be more willing and able to use technology). However, older patients were included in the pilot study—the average age in the study sample was 74 years. Given that old age is one of the factors associated with less successful self-management, it is important that this intervention aimed at improving self-management was trialed within this population. Another limitation in this study is the absence of controls. Pre-post studies like this pilot study are susceptible to regression to the mean due to the absence of appropriate controls. Regression to the mean highlights the implications of unexplained fluctuations in patient outcomes that are not attributable to the treatment itself; it spotlights the real reasons those fluctuations occur, such as patient adaptation or simple randomness [18]. However, there is a paucity of evidence on the effect of regression to the mean on economic evaluations.

Additionally, this cost analysis is based on self-report of patients and thus is prone to recall bias. The recall period in this pilot study is relatively short to minimize this bias; however, administrative data were available to improve accuracy. This study also does not account for any cost to the patient of using the TEC4Home technology, such as time spent reporting biometric data daily. However, reviews of home monitoring technologies in patients with HF did not provide insights into possible additional costs that patients might incur from using the technology [2]. The follow-up period in this feasibility study is only 90 days and the effect of the intervention on health outcomes and costs will extend long beyond the observation period and these costs will not be captured as part of this analysis. Despite these potential limitations, this study effectively achieved its objectives of detecting potential benefits of the home monitoring technology and providing information on necessary changes and refinements to the larger clinical trial, which can effectively address these limitations, given the proposed 12-month follow-up period and significantly larger sample size. Additionally, the cost analysis contributes to the literature by analyzing direct health care costs incurred by patients that are often ignored.

### Conclusion

The CCA showed positive trends in cost savings in the TEC4Home pilot study. Analysis from the health system perspective and the partial societal perspective showed statistically significant cost savings for patients enrolled in the TEC4Home arm. In line with the advantages of conducting an economic analysis alongside a feasibility study, this study facilitated the piloting of patient questionnaires and informed the methodology for the full clinical trial, which is currently underway in British Columbia, Canada.

## Abbreviations

CCA: cost-consequence analysis
ED: emergency department
GP: general practitioner
HF: heart failure
TEC4Home: Telehealth for Emergency-Community Continuity of Care Connectivity via Home-Telemonitoring
